# Supplementary material for: MANF antagonizes nucleotide exchange by the endoplasmic reticulum chaperone BiP
Source: Nat Commun. 2019 Feb 1;10:541. doi: 10.1038/s41467-019-08450-4 (PMC6358605; doi:10.1038/s41467-019-08450-4)
Supplement: Supplementary file 1 — Supplementary Information [file 41467_2019_8450_MOESM1_ESM.pdf]

MANF antagonizes nucleotide exchange by the endoplasmic reticulum (ER) chaperone BiP

Yan and Rato et al.

Supplementary Information

Supplementary Figure 1

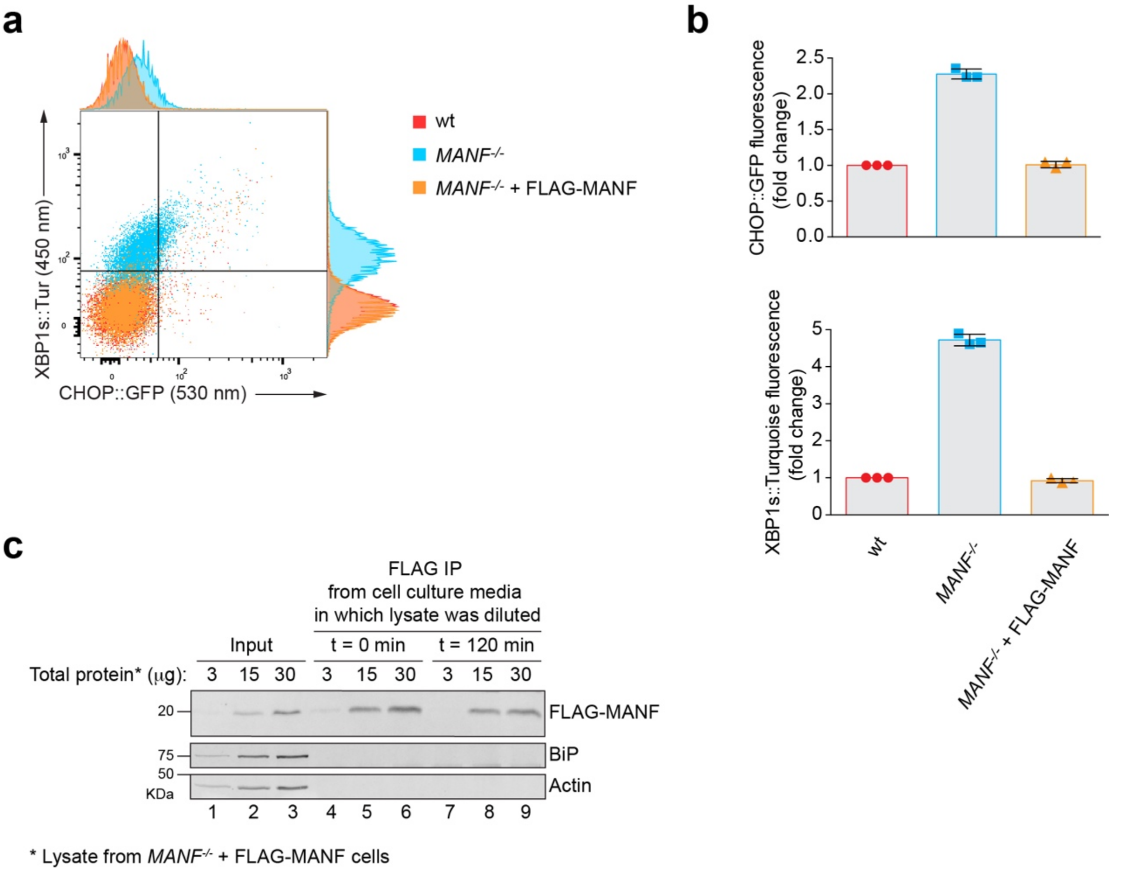

Rescue of the MANF deletion phenotype by stably expressing FLAG-M1-tagged MANF (supplementary data for Fig. 1)

a Flow cytometry analysis of CHO-K1 S21 wildtype (wt) and *MANF*$^{-/-}$ cells and *MANF*$^{-/-}$ cells stably-expressing FLAG-M1-MANF.

b Bar diagram showing the fold change relative to wt of the median values ± SD of the GFP and Turquoise fluorescence signals from three independent experiments (similar results were obtained with three independent clones). Note that the UPR reporter activation in *MANF*$^{-/-}$ cells is restored to basal levels in cells stably expressing FLAG-M1-MANF. Color code as in “a”.

c SDS-PAGE and immunoblot analysis of the FLAG-M1-MANF content of lysates from CHO-K1 S21 *MANF*$^{-/-}$ cells stably expressing FLAG-M1-MANF (3, 15 and 30 µg of total protein). Lanes 1-3 report on the FLAG-M1-MANF content of the lysate whereas lanes 4-9 are of the FLAG-M1-MANF recovered by FLAG-M1 immunoprecipitation from samples in which the lysate was diluted into cell culture media, and incubated for 0 or 120 minutes at 37˚C. Equal volumes of the immunoprecipitation samples (FLAG IP) and samples of the cell lysates (Input) were loaded. Note the efficiency of the FLAG-M1 immunoprecipitation and stability of FLAG-M1-MANF in the cell culture media. Together these findings argue that the weak MANF signal observed in the culture supernatant in Fig. 1d is an indication that most of the MANF remains intracellular. Uncropped images of the blots are presented in Supplementary Dataset 1.

Source data for panel “b” and uncropped images for panel “c” are provided as a Source Data file.

Supplementary Figure 2

a

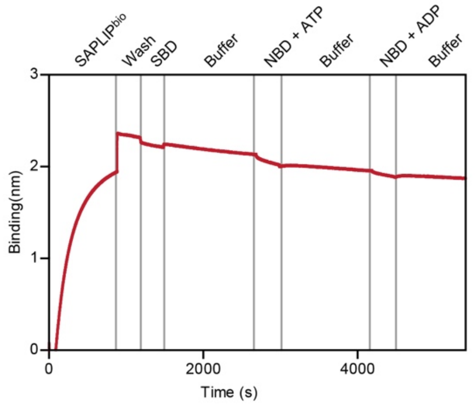

b

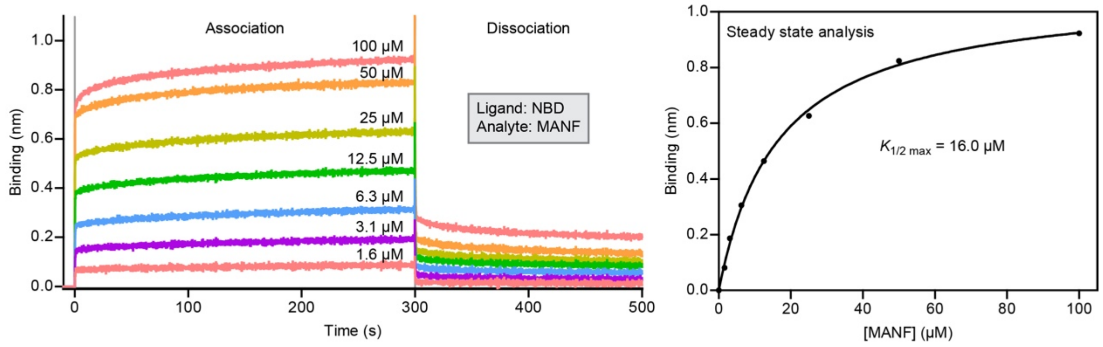

c

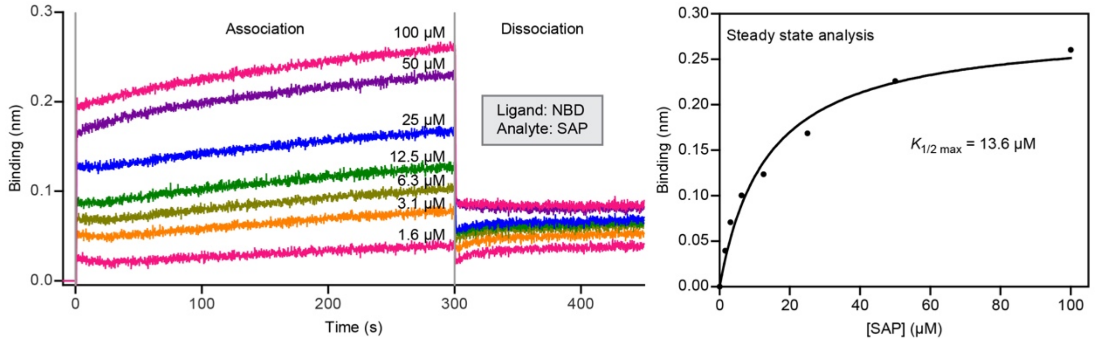

Supplementary BLI traces for Fig. 2

a Bio-layer interferometry (BLI) signals of streptavidin biosensors loaded with biotinylated SAPLIP (the first phase shown) and sequentially incubated with the BiP SBD or NBD. Note the absence of a binding signal.

b Traces of time-dependent BLI signal from a representative experiment shown in Fig. 2e of biosensors loaded with biotinylated BiP NBD and exposed to solutions containing the indicated concentrations of MANF to record association, and then transferred into buffer for dissociation. Association signals at 300 seconds were used to create the binding curve to the right. The $K_{1/2\ max}$ value was extracted by fitting the data to a saturated one site specific binding function in Prism 5.

c As in “b” above but with SAP in solution.

Supplementary Figure 3

a

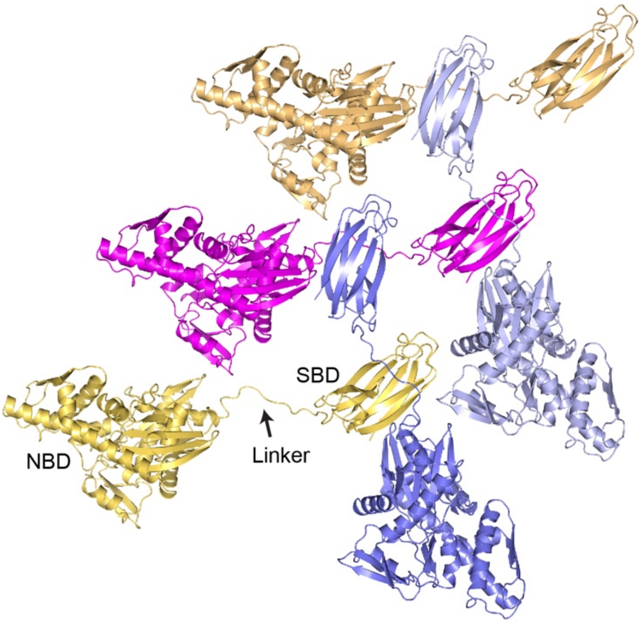

b

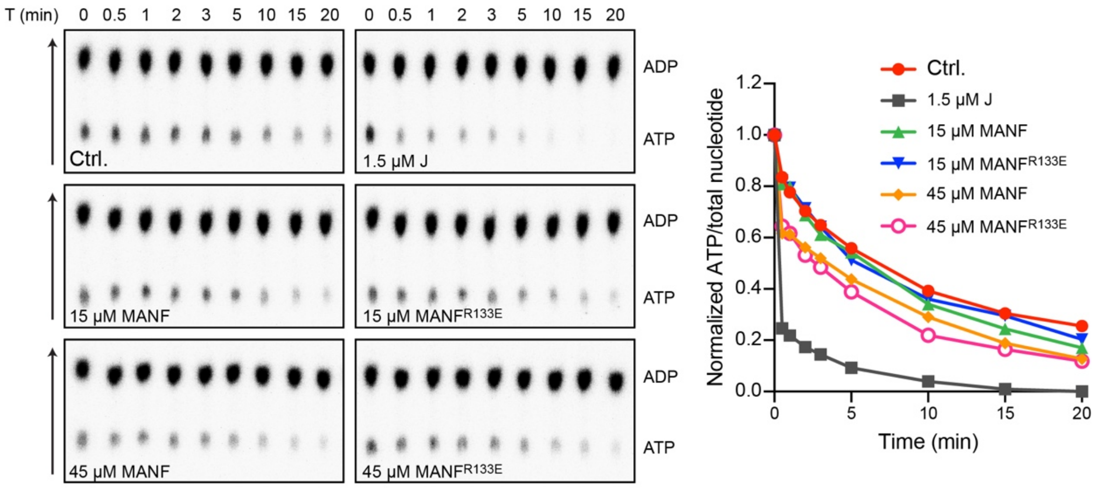

Crystal structure of BiP oligomers

a Cartoon representation of BiP molecules in the crystal of apo BiP^{V461F} (PDB 6HAB). Though a single BiP molecule was found in the asymmetric unit, in the crystal the substrate binding domain (SBD) of a symmetry-related molecule binds the interdomain linker of another molecule, forming “daisy-chain” oligomers. The nucleotide binding domain (NBD), SBD, and the interdomain linker of one BiP protomer are annotated.

b Shown is a representative autoradiograph (one of two experiments performed) of ^{32}P-labeled ATP and ADP separated by thin layer chromatography, the products of a single-turnover ATPase assay testing if MANF has a stimulatory effect on ATP hydrolysis by BiP. Pre-formed complexes between purified BiP protein and α-^{32}P-ATP

were incubated in the absence of additional proteins (control) or in the presence of the indicated concentrations of MANF, its derivatives, or the J-domain of ERdJ6 as a positive control. ATPase activity was assessed by comparing the loss of ATP signal over time and the signals were quantified in the plot to the right. Of note, the ADP signal present at t = 0 arises from a combination of factors: non-enzymatic hydrolysis of the (unlabelled) γ phosphate during storage of the precursor ^{32}P α-labelled ATP, enzymatic hydrolysis during formation of the BiP-ATP complex and possibly hydrolysis that occurs during sample freezing and thawing. However, as this is a single turnover experiment and only preformed BiP-ATP complexes can hydrolyze ATP, BiP-ADP complexes are inert and the pre-experimental conversion of ATP to ADP is ignored and only the ATPase activity of BiP-ATP complexes during the experiment are taken into account.

Supplementary Figure 4

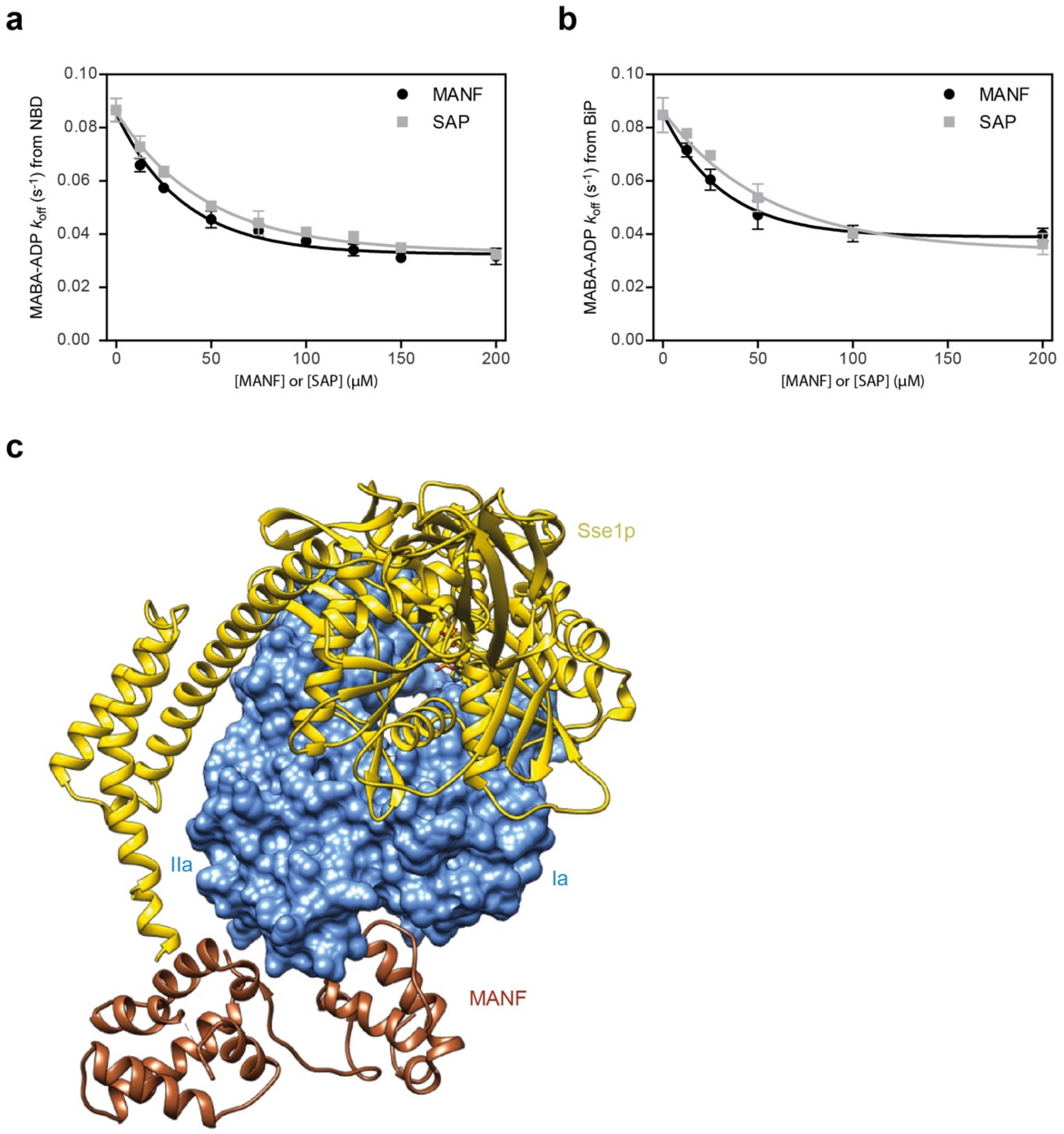

Supplementary data for Fig. 5

a Plot of k_{off} for release of MABA-ADP from BiP NBD (as in Fig. 5b) against final concentration of MANF or SAP. The mean values and SD bars of three independent experiments are plotted. Single exponential best fit lines are shown.

b As in “a” but with intact BiP and ATP as competitor. Related to Fig. 5c.

c Overlay of the NBD-MANF complex structure (as in Fig. 3a) and the Sse1p-Hsp70 complex (a nucleotide exchange factor bound to an Hsp70 NBD; PDB 3D2F). MANF (gold) binds to the opposite site of the NBD (blue surface) where the NEF Sse1p (yellow) binds.

Source data for panels “a” and “b” are provided as a Source Data file.

Supplementary Figure 5

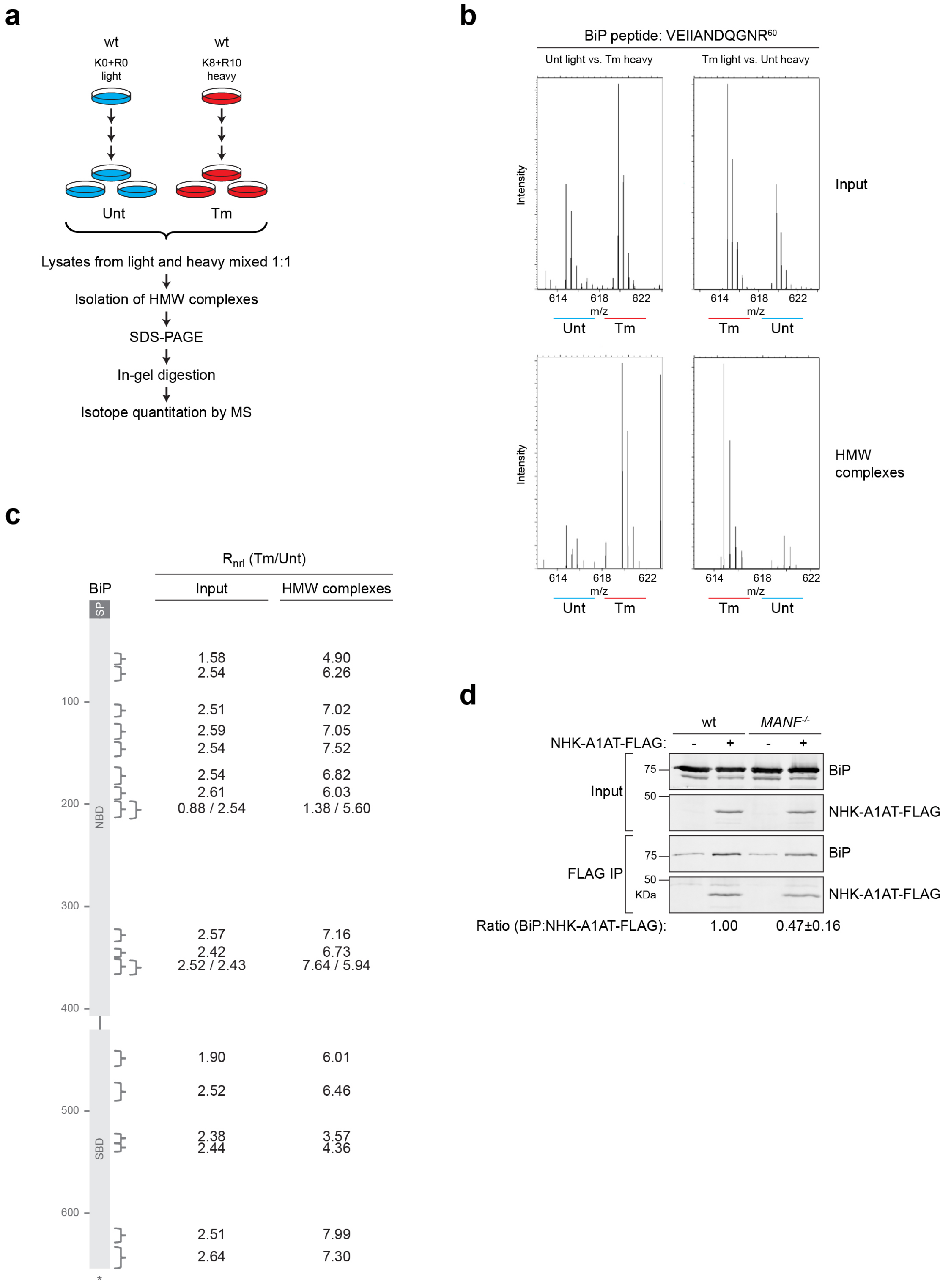

More BiP is recovered in high molecular weight complexes from tunicamycin-treated cells (supplementary data for Fig. 6)

a Schema of the design of the SILAC experiment to quantify relative changes in abundance of BiP peptides incorporated into detergent insoluble high molecular weight

(HMW) complexes in CHO-K1 S21 wildtype (wt) cells untreated and treated with tunicamycin (Tm; 2.5 μg/mL, 15 hours).

b LC-MS spectra of a representative doubly-charged tryptic BiP peptide (VEIIANDQGNR[60]) from the input (top) and HMW complexes (bottom) of experiments outlined in “a”. The spectrum on the left is from lysate of untreated (Unt) cells cultured in light medium combined with lysate from cells cultured in heavy medium and exposed to tunicamycin (Tm), and the spectrum on the right is of BiP from untreated cells cultured in heavy medium combined with lysate from cells cultured in light medium and exposed to tunicamycin.

c Averaged normalized ratios (R_{nrl}) of BiP peptides identified in the LC-MS spectra from the tunicamycin-treated cells versus untreated cells in the input and HMW complexes fraction from the two experiments as described in “b”. The position of the peptides on the BiP sequence (654 amino acids) is indicated by the brackets. The BiP signal peptide (SP), nucleotide binding domain (NBD), and substrate binding domain (SBD) are indicated.

d Immunoblot of BiP and FLAG-tagged null Hong Kong variant of α1-antitrypsin (A1AT-NHK-FLAG) in lysates of transfected CHO-K1 S21 wildtype (wt) and *MANF*$^{-/-}$ cells (Input) or recovered in an anti-FLAG immunoprecipitation (FLAG IP). The recovery of BiP, normalized to the α1-antitrypsin signal in the immunoprecipitation, is noted and is set to 100% in the sample. Shown is a representative of five experiments. The mean ratio of BiP:A1AT-NHK-FLAG recovered in the FLAG IP is provided (n = 5).

Source data for panels “c” and “d” and uncropped image for panel “d” are provided as a Source Data file.

Supplementary Figure 6

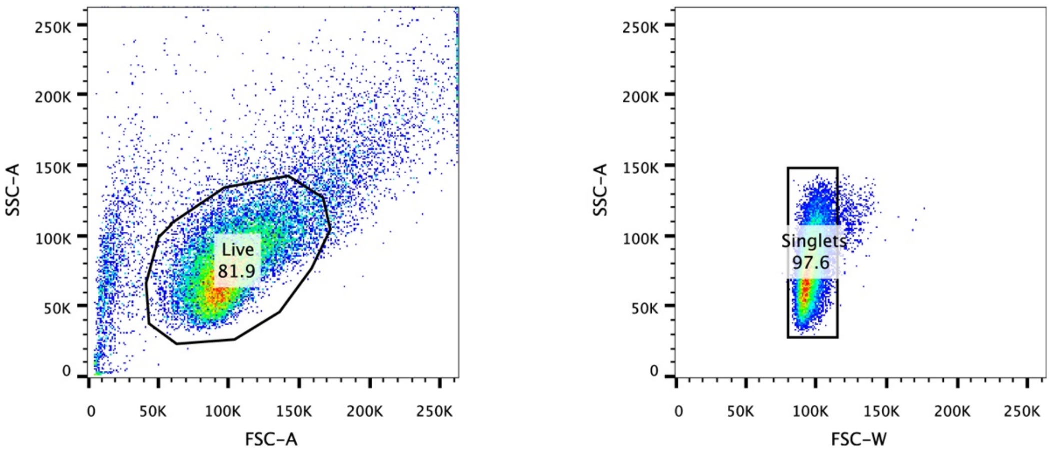

Example of the flow cytometry gating strategy

Preliminary gating for live cells was done based on FSC-A/SSC-A and for singlets based on FSC-W/SSC-A.

Supplementary Table 1

List of plasmids used, their lab names, description, first appearance in the figures and their corresponding label, and references.

ID	Plasmid name	Description	Reference	Appearances	Label in figure
UK173	haBiP_27-654_pQE10	Bacterial expression of His6-tagged wildtype hamster BiP	PMID: 18923430	Figure 5, S5	BiP
UK185	mP58(384-470)_pGEX-4T1	Bacterial expression of mouse ERdj6 (P58) J-domain fused to GST	PMID: 18923430	Figure S3B	J
UK857	haBiP_417-654_pCA528	Bacterial expression of Smt3-SBD	PMID: 26673894	Figure 2D, S2A	SBD
UK1610	pSpCas9(BB)-2A-mCherry_V2	Mammalian expression of Cas9 from *S. pyogenes* with 2A-mCherry and cloning backbone for sgRNA	PMID: 27918543		
UK1825	haBiP_27-654_T229A_V461F_pQE10	Bacterial expression BiP 27-654 with both T229A and V461F mutations	PMID: 29064368	Figure 2C	$BiP^{T229A\text{-}V461F}$
UK1839	cgMANF_g1_pSpCas(BB)-2A-mCherry	mCherry-tagged CRISPR (UK1610) for Chinese targeting hamster MANF gene (sgRNA sequence including PAM: GCTACAGTGCTACTACATTGGGG)	This study		
UK1840	cgMANF_g2_pSpCas(BB)-2A-mCherry	mCherry-tagged CRISPR (UK1610) for targeting Chinese hamster MANF gene (sgRNA sequence including PAM: GGATACCTCATTGATGATCTTGG)	This study		
UK1987	mMANF_22-179_pGEX_TEV_AviTag	Bacterial expression of GST-TEV- mature mouse MANF with N-term AviTag	This study	Figure 2C, 2D	ligand MANF
UK2004	mMANF_22-123_pGEX_TEV_AviTag	Bacterial expression of GST-TEV- mouse MANF N-term SAPLIP domain with N-terminal AviTag	This study	Figure S2A	ligand SAPLIP
UK2005	mMANF_119-179_pGEX_TEV_AviTag	Bacterial expression of GST-TEV- mouse MANF N-term SAP domain with N-terminal AviTag	This study	Figure 2C, 2D	ligand SAP
UK2006	Smt3_mMANF_22-179_pET-21a	Bacterial expression of Smt3-tagged authentic mMANF	This study	Figure 2E, S2B, 3, 4B, 4C, 4D, 5, S5	MANF
UK2013	mMANF_22-123_pGEX_TEV MP3	Bacterial expression of mMANF SAPLIP	This study	Figure 5	
UK2022	haBiP_19-413_AviTag_pCA528	Bacterial expression Smt3-tagged authentic haBiP NBD with C-term AviTag	This study	Figure 2E, S2B, S2C, 4C, 4D	NBD-bio
UK2039	Smt3-haBiP_28-413_pQE30	Bacterial expression of Smt3-NBD	This study	Figure 2D, 3,4A, 4B	NBD
UK2058	pBABEpu_FLAGM1	pBABE with a signal peptide and C-terminal FLAG-M1. Puromycin-resistance.	This study		
UK2059	pBABEpu_FLAGM1_mMANF_22-179	Mammalian expression of FLAG-M1-tagged mouse MANF (22-179) (UK2058 backbone)	This study	Figure 1D, 2A, S1A, S1B, S1C	FLAG-MANF
UK2079	Smt3-mMANF_126-169_pSUMO3	Bacterial expression of Smt3-tagged mMANF SAP	This study	Figure 2E, S2C, 3 A, 4A, 5, S5	SAP
UK2121	Smt3 -haBiP_28-549_V461F_pQE30	Bacterial expression of Smt3_haBiP_28-549_V461F	This study	Figure 3D, S3	BiP^{V461F}
UK2209	Smt3-mMANF_22-179_pET-21a-R133E	MANF mutagenesis R133E, based on UK2006	This study	Figure 4D, 5	R133E
UK2210	Smt3-mMANF_22-179_pET-21a-E153A	MANF mutagenesis E153A, based on UK2006	This study	Figure 4D, 5	E153A
UK2212	Smt3-mMANF_22-179_pET-21a-K138A	MANF mutagenesis K138A, based on UK2006	This study	Figure 4D	K138A
UK2225	huORP150_33-999_pCA833	Bacterial expression of H6-Smt3 human ORP150 Grp170 (from Claes Andreasson)	This study	Figure 5D	Grp170
UK2280	Smt3-mMANF_22-179_pET-21a-R23A	MANF mutagenesis R23A, based on UK2006	This study	Figure 4D	R23A
UK2283	A1AT_NHK_QQQ_pCDNA5_FRT_TO_3XFLAG	Mammalian expression of C-terminally FLAG-M2-tagged null Hong Kong alpha1-antitrypsin null Hong Kong with mutation NNN to QQQ (derived from hA1AT-NHK-QQQ-pREP9 (PMID 16629899), a gift of Nobuko Hosokawa, University of Kyoto.	This study	Figure S5D	NHK-A1AT-FLAG

**Supplementary Table 2**			
**List of primers used in this study.**			
**Primer ID**	**Primer name**	**Sequence (5' to 3')**	**Plasmid constructed (ID)**
1565	cgMANF_g1_S	CACCGGCTACAGTGCTACTACATTG	UK1839
1566	cgMANF_g1_AS	AAACCAATGTAGTAGCACTGTAGCC	UK1839
1567	cgMANF_g2_S	CACCGGGATACCTCATTGATGATCT	UK1840
1568	cgMANF_g2_AS	AAACAGATCATCAATGAGGTATCCC	UK1840
1785	mMANF_BamHI_22S	GACAGCGGATCCCTGCGGCCAGGAGACTGTGAAG	UK1987
1786	mMANF_HD3_AS	ATTGGGAAGCTTACAGATCAGTCCGTGCGCTGG	UK1987
3	pGEX5'	GGGCTGGCAAGCCACGTTTGGTG	UK2004
1820	mMANF_D123_HD3_AS	TCCGCTAAGCTTTAGTCAATCTGCTTGTCGTATTTTAG	UK2004
1821	mMANF_D119_BamHI_S	GAACTAGGATCCGACAAGCAGATTGACCTGAGC	UK2005
4	pGEX3'	CCGGGAGCTGCATGTGTCAGAGG	UK2005
1837	haBiP_1837	CAACAGAGCTGTGCAGAAACTTCG	UK2022
1838	haBiP_D413_AviTag	GCCGCCAAGCTTATTCATGCCATTCAATTTTCTGTGCCTCGAAGATGTCATTCAAACCATCACCTGTATCTTGATCACCAGA	UK2022
629	pGEX_Distal_AS	GCACATTTCCCCGAAAAGTGCCA	UK2039
1839	haBiP_D413*	GCCGCCAAGCTTAATCACCTGTATCTTGATCACCAGA	UK2039
97	CMVf	CGCAAATGGGCGGTAGGCGTG	UK2058
440	FLAG_Seq_hGH_2AS	GCACTGGAGTGGCAACTTCC	UK2058
1831	mMANF_EcoRI_22_S	GTGGTGAATTCACTGCGGCCAGGAGACTGTG	UK2059
1902	mMANF_XbaI_SalI_179_AS	ACCACGTCGACTCTAGACTACAGATCAGTCCGTGCGC	UK2059
1918	mMANF-126-BamH1-f	GACAGGGATCCACAGTGGACCTGAAG	UK2079
1919	mMANF-169-Hind3-r	CTTCAAGCTTAGGCGTATTTAGGCAT	UK2079
2085	MANF-R133E-f	GACCTGAAGAAGCTCgaGGTGAAAGAGCTGAAG	UK2209
2086	MANF-R133E-r	CTTCAGCTCTTTCACCtcGAGCTTCTTCAGGTC	UK2209
2089	MANF-E153A-f	TGCAAAGGCTGTGCAGcAAAGTCTGACTATATC	UK2210
2090	MANF-E153A-r	GATATAGTCAGACTTTgCTGCACAGCCTTTGCA	UK2210
2087	MANF-K138A-f	CGGGTGAAAGAGCTGgcGAAGATCCTGGACGAC	UK2212
2088	MANF-K138A-r	GTCGTCCAGGATCTTCgcCAGCTCTTTCACCCG	UK2212
2191	mMANF_R23A_1F	TGGTGGTCTGgcGCCAGGAGAC	UK2280
2192	mMANF_R23A_1R	ATCTGTTCTCTGTGAGCC	UK2280
603	A1AT_NHK_HD3_S	GCTGCTAAGCTTGCCATGCCGTCTTCTGTCTCGTG	UK2283
604	A1AT_NHK_XhoI	GGGCTGCTCGAGTGCACGGCCTTGGAGAGCTTCAG	UK2283

Supplementary Table 3

Data collection and refinement statistics.

	NBD-SAP	NBD-MANF	BiP^{V461F} (apo)
**Data collection**			
Synchrotron stations	DLS I04	DLS I04-1	DLS I04-1
Space group	C121	P1	$P12_11$
a,b,c; Å	153.31, 66.75, 44.30	58.42, 60.96, 96.11	50.61, 52.50, 91.73
α, β, γ; °	90.00, 106.54, 90.00	81.13, 88.33, 74.33	90.00, 97.34, 90.00
Resolution, Å*	42.47-1.57 (1.61-1.57)	36.52-2.49 (2.55-2.49)	50.2-2.08 (2.13-2.08)
R_{merge}*	0.054 (0.860)	0.05 (0.707)	0.029 (0.775)
$\langle I/\sigma(I) \rangle$*	11.3 (1.2)	13.3 (1.6)	16.5 (1.4)
$CC_{1/2}$*	0.998 (0.520)	0.999 (0.617)	1 (0.602)
No. of unique reflections*	59404 (4405)	43348 (3227)	28693 (2082)
Completeness, %*	99.3 (99.8)	98.1 (98.1)	99.2 (99.5)
Redundancy*	3.3 (3.3)	3.5 (3.6)	3.3 (3.2)
**Refinement**			
R_{work}/R_{free}	0.184 / 0.204	0.226 / 0.256	0.238 / 0.271
No. of atoms (non H)	3591	8235	3755
Average B-factors	25.1	68.3	52.1
RMS Bond lengths Å	0.007	0.004	0.002
RMS Bond angles,°	1.225	0.794	1.208
Ramachandran favored region, %	99.5	99.5	97.5
Ramachandran outliers, %	0	0	0
MolProbity score†	0.87 (100th)	0.85 (100th)	1.25 (100th)
PDB code	6H9U	6HA7	6HAB
PDB code	6H9U	6HA7	6HAB

* Values in parentheses are for highest-resolution shell.
†100th percentile is the best among structures of comparable resolutions. 0th percentile is the worst.
