# Supplementary material for: MANF antagonizes nucleotide exchange by the endoplasmic reticulum chaperone BiP
Source: Nat Commun. 2019 Feb 1;10:541. doi: 10.1038/s41467-019-08450-4 (PMC6358605; doi:10.1038/s41467-019-08450-4)
Supplement: Supplementary file 3 — Description of Additional Supplementary Files [file 41467_2019_8450_MOESM3_ESM.pdf]

Description of Additional Supplementary Files

File Name: Supplementary Movie 1
Description: Morph animation of BiP's transitioning from a model of the domain-undocked BiP (PDB 6HAB) with a superimposed molecule of MANF (derived from a structure of the NBD-MANF complex; PDB 6HA7) to the domain-docked state of BiP (PDB 5E84). It reveals a steric incompatibility between MANF binding and the transition to the domain-docked conformation assumed by the ATP-bound BiP. The nucleotide binding domain (NBD) of BiP and MANF from the NBD-MANF complex structure are shown in purple and gold, respectively. The substrate binding domain (SBD) of BiP is shown in pink, its interdomain linker in red, and its NBD subdomains are presented in grey (Ia), green (Ib), blue (IIa), and yellow (IIb).
