# Supplementary material for: MANF antagonizes nucleotide exchange by the endoplasmic reticulum chaperone BiP
Source: Nat Commun. 2019 Feb 1;10:541. doi: 10.1038/s41467-019-08450-4 (PMC6358605; doi:10.1038/s41467-019-08450-4)
Supplement: Supplementary file 6 — Reporting Summary [file 41467_2019_8450_MOESM6_ESM.pdf]

nature research

Corresponding author(s): David Ron and Steffen Preissler

Reporting Summary

Nature Research wishes to improve the reproducibility of the work that we publish. This form provides structure for consistency and transparency in reporting. For further information on Nature Research policies, see Authors & Referees and the Editorial Policy Checklist.

Statistical parameters

When statistical analyses are reported, confirm that the following items are present in the relevant location (e.g. figure legend, table legend, main text, or Methods section).

n/a	Confirmed	
☐	☒	The exact sample size (*n*) for each experimental group/condition, given as a discrete number and unit of measurement
☐	☒	An indication of whether measurements were taken from distinct samples or whether the same sample was measured repeatedly
☐	☒	The statistical test(s) used AND whether they are one- or two-sided<br>*Only common tests should be described solely by name; describe more complex techniques in the Methods section.*
☒	☐	A description of all covariates tested
☒	☐	A description of any assumptions or corrections, such as tests of normality and adjustment for multiple comparisons
☐	☒	A full description of the statistics including central tendency (e.g. means) or other basic estimates (e.g. regression coefficient) AND variation (e.g. standard deviation) or associated estimates of uncertainty (e.g. confidence intervals)
☐	☒	For null hypothesis testing, the test statistic (e.g. *F*, *t*, *r*) with confidence intervals, effect sizes, degrees of freedom and *P* value noted<br>*Give P values as exact values whenever suitable.*
☒	☐	For Bayesian analysis, information on the choice of priors and Markov chain Monte Carlo settings
☒	☐	For hierarchical and complex designs, identification of the appropriate level for tests and full reporting of outcomes
☒	☐	Estimates of effect sizes (e.g. Cohen's *d*, Pearson's *r*), indicating how they were calculated
☐	☒	Clearly defined error bars<br>*State explicitly what error bars represent (e.g. SD, SE, CI)*

Our web collection on statistics for biologists may be useful.

Software and code

Policy information about availability of computer code

Data collection	Flow cytometry data were acquired using FACSDivaA (BD Bioscience)<br><br>Crystallographic data collection was done at the Diamond Light Source through the GDA (Generic Data Acquisition) software.
Data analysis	Mass spectrometry data were analysed with Maxquant 1.5.8.3<br><br>Statistical analyses were performed with Prism 5 (GraphPad)<br><br>Flow cytometry data were analysed using FlowJo X 10.0.7r2<br><br>Crystallographic data was processed by the XIA2 pipeline implementing Mosflm or XDS for indexing and integration, Pointless for space group determination, and Aimless for scaling and merging. All structures were solved by molecular replacement using Phaser. Manual building was performed with COOT. Further refinement was performed iteratively using COOT, phenix.refine and refmac5. MolProbity was consulted throughout the refinement process. Molecular graphics were generated using UCSF Chimera and PyMol (The PyMOL Molecular Graphics System, Version 1.3 Schrödinger, LLC).

For manuscripts utilizing custom algorithms or software that are central to the research but not yet described in published literature, software must be made available to editors/reviewers upon request. We strongly encourage code deposition in a community repository (e.g. GitHub). See the Nature Research guidelines for submitting code & software for further information.

Data

Policy information about availability of data

All manuscripts must include a data availability statement. This statement should provide the following information, where applicable:

- Accession codes, unique identifiers, or web links for publicly available datasets
- A list of figures that have associated raw data
- A description of any restrictions on data availability

Atomic coordinates of the X-ray structures were deposited in the Protein Data Bank with accession codes 6H9U (NBD-SAP), 6HA7 (NBD-MANF), and 6HAB (BiPV461F).

Field-specific reporting

Please select the best fit for your research. If you are not sure, read the appropriate sections before making your selection.

☒ Life sciences ☐ Behavioural & social sciences ☐ Ecological, evolutionary & environmental sciences

For a reference copy of the document with all sections, see nature.com/authors/policies/ReportingSummary-flat.pdf

Life sciences study design

All studies must disclose on these points even when the disclosure is negative.

Sample size	No statistical methods were used to determine samples size.
Data exclusions	No data were excluded.
Replication	Number of independent replicates are stated in the corresponding figure legends.
Randomization	Not relevant to this study.
Blinding	Not relevant to this study.

Reporting for specific materials, systems and methods

Materials & experimental systems

n/a	Involved in the study
☒	☐ Unique biological materials
☐	☒ Antibodies
☐	☒ Eukaryotic cell lines
☒	☐ Palaeontology
☒	☐ Animals and other organisms
☒	☐ Human research participants

Methods

n/a	Involved in the study
☒	☐ ChIP-seq
☐	☒ Flow cytometry
☒	☐ MRI-based neuroimaging

Antibodies

Antibodies used

Primary antibodies against hamster BiP (chicken anti-BiP), actin (mouse anti-actin), MANF (chicken anti-MANF), FLAG-M1 (mouse ANTI-FLAG-M1), and A1AT (mouse anti-A1AT) were used.

Validation

chicken anti-BiP: Antibody was described in Avezov, E. et al. Lifetime imaging of a fluorescent protein sensor reveals surprising stability of ER thiol redox. J Cell Biol 201, 337-49 (2013)

mouse anti-actin: Abcam, cat. # AB3280

chicken anti-MANF: Validated by comparing wild-type and MANF knockout cells (Fig. 1b in this study) and by immunoblotting against dilutions of the antigen (data available upon request).

mouse ANTI-FLAG-M1: Sigma, cat. # F3040

	mouse anti-A1AT monoclonal: Abcam, cat. # AB9399

Eukaryotic cell lines

Policy information about cell lines

Cell line source(s)	CHO-K1 S21, CHP-K1 S21 MANF-/-, CHP-K1 S21 MANF-/- stably expressing FLAG-MANF
Authentication	The cells were phenotypically validated as proline auxotrophs and their Cricetulus griseus origin was confirmed by genomic sequencing.
Mycoplasma contamination	Cell lines were subjected to random testing for mycoplasma contamination using the MycoAlert Mycoplasma Detection Kit (Lonza).
Commonly misidentified lines (See ICLAC register)	No misidentified cell lines were used.

Flow Cytometry

Plots

Confirm that:

☒ The axis labels state the marker and fluorochrome used (e.g. CD4-FITC).

☒ The axis scales are clearly visible. Include numbers along axes only for bottom left plot of group (a 'group' is an analysis of identical markers).

☒ All plots are contour plots with outliers or pseudocolor plots.

☒ A numerical value for number of cells or percentage (with statistics) is provided.

Methodology

Sample preparation	The effect of MANF-deficiency on UPR signalling was analysed by flow cytometry. The sensitivity to UPR induction was tested by treating cells with the UPR-inducing compound, thapsigargin, for 16 hours before analysis. Cells were washed with PBS and collected in PBS containing 4 mM EDTA, and single cell fluorescence signals were analysed by dual-channel flow cytometry. GFP and mCherry fluorescence was detected with excitation laser 488 nm, filter 530/30, and excitation laser 561, filter 610/20, respectively.
Instrument	LSRFortessa cell analyzer (BD Biosciences)
Software	Flow cytometry data were collected using FACSDivaA (BD Bioscience)<br><br>Flow cytometry data were analysed using FlowJo and Prism 5
Cell population abundance	20,000 cells per sample were analysed
Gating strategy	Preliminary gating for live cells was done based on FSC-A/SSC-A.<br>Gating for singlets was done based on FSC-W/SSC-A.

☒ Tick this box to confirm that a figure exemplifying the gating strategy is provided in the Supplementary Information.
